# Balance Dysfunction in Parkinson's Disease: The Role of Posturography in Developing a Rehabilitation Program

**DOI:** 10.1155/2015/520128

**Published:** 2015-10-04

**Authors:** Davide Ferrazzoli, Alfonso Fasano, Roberto Maestri, Rossana Bera, Grazia Palamara, Maria Felice Ghilardi, Gianni Pezzoli, Giuseppe Frazzitta

**Affiliations:** ^1^Department of Parkinson Disease and Brain Injury Rehabilitation, “Moriggia-Pelascini” Hospital, Gravedona ed Uniti, Italy; ^2^Morton and Gloria Shulman Movement Disorders Clinic and the Edmond J. Safra Program in Parkinson's Disease, Division of Neurology, Toronto Western Hospital, UHN, University of Toronto, Toronto, ON, Canada; ^3^Department of Biomedical Engineering, Scientific Institute of Montescano, S. Maugeri Foundation, IRCCS, Montescano, Italy; ^4^Department of Physiology, Pharmacology & Neuroscience, CUNY Medical School, New York, NY 10031, USA; ^5^Parkinson Institute, Istituti Clinici di Perfezionamento, Milano, Italy; ^6^Fondazione Europea Ricerca Biomedica (FERB), “S.Isidoro” Hospital, Trescore Balneario, Italy

## Abstract

Balance dysfunction (BD) in Parkinson's disease (PD) is a disabling symptom, difficult to treat and predisposing to falls. The dopaminergic drugs or deep brain stimulation does not always provide significant improvements of BD and rehabilitative approaches have also failed to restore this condition. In this study, we investigated the suitability of quantitative posturographic indicators to early identify patients that could develop disabling BD. Parkinsonian patients not complaining of a subjective BD and controls were tested using a posturographic platform (PP) with open eyes (OE) and performing a simple cognitive task [counting (OEC)]. We found that patients show higher values of total standard deviation (SD) of body sway and along the medio-lateral (ML) axis during OE condition. Furthermore, total and ML SD of body sway during OE condition and total SD of body sway with OEC were higher than controls also in a subgroup of patients with normal Berg Balance Scale. We conclude that BD in Parkinsonian patients can be discovered before its appearance using a PP and that these data may allow developing specific rehabilitative treatment to prevent or delay their onset.

## 1. Introduction

Parkinson's disease (PD) is one of the most common neurological disorders and balance dysfunction (BD) is a common feature [1]. BD is a highly disabling symptom, difficult to treat, and predisposing patients to unexpected falls [2]. The uses of dopaminergic drugs or deep brain stimulation do not provide significant improvements of BD, probably due to a neuropathological process spreading towards nondopaminergic pathways [3].

Moreover, previous studies have demonstrated that treatment with levodopa increases postural sway in patients with advanced PD [4]. BD is characterized by alterations of postural control strategies during standing tasks responding to an unexpected destabilizing perturbation or performing voluntary movements [5]. From the stage 2 Hoehn and Yahr Scale [6], Parkinsonian patients stand with an increasingly narrow stance and stooped posture [7]. The increased muscle tone in flexor muscles and an impaired proprioception, modifying the sense of position, contribute to this posture [8], which leads to a displacement of the body center of mass over the base of support [7]. Because of that, Parkinsonians in quiet stance show an alteration of the physiological postural sways consisting of higher velocity and frequency compared to healthy controls [7]. Since medical and surgical treatments do not have beneficial effects on these symptoms, it is necessary to look for other intervention strategies.

There is an increasing body of evidence, confirmed by systematic reviews [9, 10], that physical therapy interventions can improve BD of people with PD [3]. Unfortunately, the small number and the limited quality of the included trials call for more research in order to clarify the real impact of exercise on balance. A large cohort of rehabilitation interventions have been proposed for BD: exercises programs with balance training components [9], external cueing training [11], treadmill training [12], training with external perturbations [1], progressive resistance exercise [13], hydrotherapy [14], dance [15], and movement strategy training [16]. Nevertheless, the optimal design and delivery of programmes remain unclear [3].

Postural sway can be abnormal in persons with PD before the onset of clinical BD symptoms [17]. This fact has important implications because probably exists the possibility to identify, quantify, and treat BD in patients who do not complain of postural instability. Posturography has been recognized as a useful technique to assess balance in PD [18]. The aim of our study is to investigate the utility of the quantitative posturographic indicators in the assessment of balance in PD patients not complaining of BD in order to develop a tailored rehabilitation treatment specifically addressing its prevention.

## 2. Methods

### 2.1. Participants

We enrolled, at the Department of Parkinson's Disease and Brain Injury Rehabilitation of Moriggia-Pelascini Hospital, Gravedona ed Uniti, Italy, twenty-nine PD patients not complaining of BD. Patients were diagnosed according to the UK Brain Bank criteria [19] and were evaluated by a neurologist specialized in movement disorders. Twelve controls matched for age and sex were recruited from our database of volunteers.

The inclusion criteria were (i) stage 2.5–3 according to the Hoehn and Yahr Scale, (ii) stable pharmacological treatment for the last 8 weeks, and (iii) Mini-Mental State Examination (MMSE) >25.

Exclusion criteria were (i) focal brain lesions, (ii) disabling drug-induced dyskinesias, (iii) disturbing tremor, and (iv) vestibular/visual dysfunction limiting balance. Patients included in stage 3 H&Y should not complain of BD in spite of impaired postural reflexes objectively found at the pull test.

All patients and controls underwent a posturography at 9 AM and during the medication ON state (in case of PD patients).

Unified Parkinson's Disease Rating Scale (UPDRS) was assessed in PD patients, while the Berg Balance Scale (BBS) [20] was assessed in both groups before posturography. The BBS is a 14-item test designed to measure the balance of older adults by assessing their performance of specific functional tasks. Each task is scored from 0 to 4, for a maximum of 56 points. A score of 41–56 is associated with a low fall risk, 21–40 with a medium fall risk, and 0–20 with a high fall risk [21].

The study design and protocol were approved by the local Scientific Committee (Moriggia-Pelascini General Hospital, Gravedona ed Uniti, Como) and were in accordance with the code of Ethics of the World Medical Association (Declaration of Helsinki, 1967). A complete explanation of the study protocol was provided to and written informed consent was obtained from all patients before the participation in the study.

### 2.2. Posturographic Platform

PD patients and controls were tested using a posturographic platform (PP) (Prokin 254 (Pro-Kin Software Stability), TecnoBody S.r.l., Dalmine, 24044 Bergamo, Italy), according to standardized methods [22]. The PP is a force platform with a flat and regular surface fixed to four force-transduction systems. The related set of signals is sent to a computer for offline analysis and is used to detect the position of the center of pressure (CoP). The CoP represents the point of application of forces exchanged between feet and ground. The CoP area is an index of the effectiveness of the tonic postural system in keeping the center of gravity closer to the intermediate position of balance.

Patients and controls were required to stand still on a force plate with their feet positioned comfortably within a box defined by dimensions equal to their foot length. They were instructed to look straight ahead at a screen surface placed 80 cm away and to keep arms comfortably at their sides during the stances in a normal forward-facing position, with eyes focused on a stationary target. Each participant performed two standing tests, each epoch lasting 30 seconds. Using the PP as a visual feedback, patients were asked to maintain a cursor sensitive to the displacement of the center of gravity, within a target located in the center of the screen. The standard deviation (SD) of body sway [total and along the anteroposterior (AP) and mediolateral (ML) axis] was calculated and expressed in mm. Total SD of body sway was defined as the mean error of the CoP on the *x*-*y* directions with respect to the trunk axis. In addition, we analyzed the statokinesigram, which is the layout of a line connecting the different positions of the CoP. Statokinesigram is not a geometrical figure and in order to quantify the dispersion of the successive CoP we used the area of the body sway: this is the area ellipse (measured in mm^2^) containing 90% of the sampled positions of the CoP. These measurements were obtained in two conditions: with open eyes (OE) and performing a simple cognitive task [counting (OEC)].

Subsequently, the posturographic data were analysed on the basis of the BBS values.

### 2.3. Statistical Analysis

The normality of the distribution of all variables was assessed by the Shapiro-Wilk statistic, supported by visual inspection. Descriptive statistics of continuous variables are reported as mean ± SD. Between-group comparisons for continuous data were assessed with unpaired *t*-test or with Mann-Whitney *U*-test in case of violation of the normality assumption. Comparisons for categorical variables were carried out by the chi-square test or Fisher's exact test when appropriate. The association between pairs of variables was assessed by the Pearson correlation coefficient. To assess the association between BBS values and measurements obtained from PP, multiple regression analysis was used, with BBS as dependent variable and posturographic parameters as predictors. Nonsignificant variables were eliminated by a backward elimination procedure at the 0.15 significance level. A *p* value <0.05 was considered statistically significant. When multiple comparisons were carried out, the Bonferroni correction was applied. Accordingly, when couples of comparisons were considered, the significance level was set to 0.025. All analyses were carried out using the SAS/STAT statistical package, release 9.2 (SAS Institute Inc., Cary, NC, USA).

## 3. Results

Demographic and clinical characteristics of patients and controls are shown in Table 1.

The values of BBS and the posturographic parameters observed in patients and controls are reported in Table 2.

Due to some skewness, several posturographic variables did not fully satisfy formal Shapiro-Wilk test for normal distribution, but violations to the normality assumption were not marked. All results obtained from parametric tests were therefore checked using also nonparametric statistics, which consistently yielded superimposable results.

**Figure 1:**
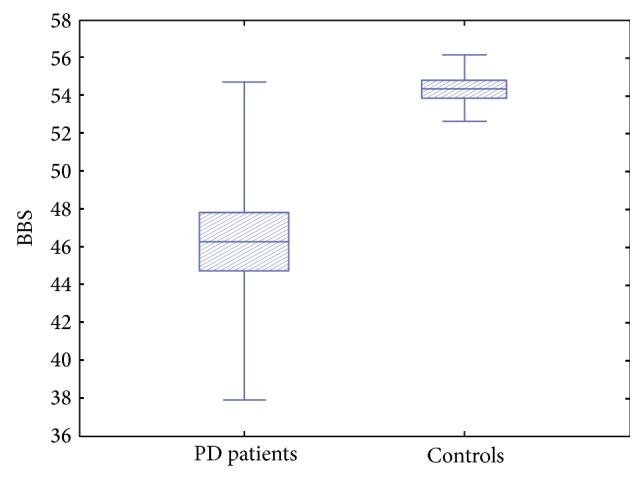
Berg Balance Scale values in PD patients compared to controls.* As expected, BBS values were lower in PD patients compared to controls.*

**Figure 2:**
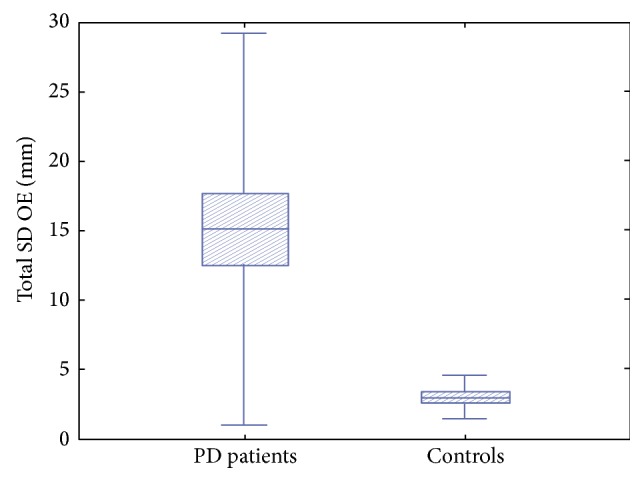
Total standard deviation of trunk sway with open eyes in PD patients with respect to controls.* PD patients showed higher values of total standard deviation (SD) of trunk sway with open eyes (OE) with respect to controls.*

**Figure 3:**
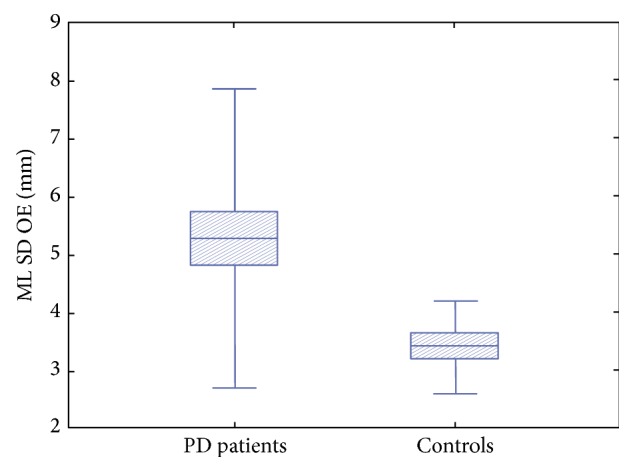
Mediolateral standard deviation of trunk sway with open eyes in PD patients with respect to controls.* PD patients showed higher values of mediolateral (ML) standard deviation (SD) of trunk sway with open eyes (OE) with respect to controls.*

As expected, BBS values were lower in PD patients compared to controls (*p* = 0.002) (Figure 1). Statistical analysis revealed significant differences in the SD of body sway between PD patients and controls. In particular PD patients showed higher values of total SD of body sway during OE (*p* = 0.005) (Figure 2) and OEC (*p* = 0.020) and along the ML axis with OE (*p* = 0.019) (Figure 3), while values along the ML axis in the OEC condition showed a trend toward higher values, but the difference did not reach statistical significance (*p* = 0.172).

Area ellipse with OE and OEC was not significantly different across groups (*p* = 0.195 and *p* = 0.297, resp.).

Focusing on the subgroups of PD patients with low risk of falls according to BBS score (BBS >40, *N* = 23), the values of ML and total SD of trunk sway with OE and total SD of trunk sway with OEC were higher than controls, with Bonferroni corrected statistical significance reached only for the last two comparisons (*p* = 0.050, *p* = 0.013, and *p* = 0.017, resp.).

The same variables were significantly higher than in controls also selecting only PD patients with BBS values comparable to controls (*N* = 11 patients with BBS > 51) (borderline significant *p* = 0.029 for ML SD of trunk sway with OE, *p* = 0.012 for total SD of trunk sway with OE, and *p* = 0.019 for total SD of trunk sway with OEC). The results of correlation analysis are listed in Tables 3(a) and 3(b) for PD patients and controls, respectively. While no association was found between BBS scores and posturographic variables in controls, in PD patients a significant negative correlation between BBS scores and ML SD of the trunk with OE (*r* = −0.49, *p* = 0.007) and with the area ellipse with OEC (*r* = −0.53, *p* = 0.004) was observed. In order to gain some insight into this finding, we performed further correlation analysis considering first the subgroup of patients at medium risk of falls (BBS ≤ 40) and then the group of patients with BBS values similar to controls (BBS ≥ 51). The finding of a strong association in the first case and of no association in the second case suggests the possibility of a ceiling effect for higher BBS values.

As expected, many posturographic parameters were significantly correlated with each other, with several values of Pearson correlation coefficient > 0.80. No significant correlation was found between posturographic parameters and disease severity as evaluated with UPDRS.

Finally, the backward variable selection procedure in multiple regression analysis revealed that ML SD of body sway with OE was an independent predictor of BBS (*p* = 0.007).

## 4. Discussion and Rehabilitation Perspectives 

### 4.1. Posturographic Findings

The main finding of our study is that, in comparison to a control group of healthy people, PD patients not complaining of BD have higher values of total SD of body sway and along the ML axis during OE condition. Total and ML SD of body sway during OE condition and total SD of body sway with OEC were higher than controls in a subgroup of Parkinsonians with low risk of falls and also in those patients with normal BBS values. Since neither disease duration nor disease severity evaluated by UPDRS were correlated with posturographic values, we can hypothesize that these are specifically related to balance control disruption and not to global disease severity, showing a high sensitivity of these posturographic parameters to early balance disturbances in PD patients not complaining of BD.

Poor balance is a typical characteristic of PD. This has already been demonstrated in advanced PD, but our data stress the need to diagnose these alterations as early as possible in order to establish a specific rehabilitative treatment. As a matter of fact, our finding of relatively higher measurements in total body sway in Parkinsonians not complaining of BD means that balance control in PD is affected even in absence of clinical signs or subjective symptoms. Our study seems to indicate that the higher body sway results mainly from increased oscillations in ML axis. Previously, increased sway in ML direction has been associated with falls in a number of conditions including PD [23]. On the contrary, several authors found that balance in the ML axis is preserved in Parkinsonians and this should explain why their gait is typically narrow-based [24] and why they have few balance problems moving sideways [25]. In this regard, patients are still able to ride a bicycle, which is an activity that requires a coordinated interplay between rhythmic pedaling and maintaining balance in the ML plane, even in the face of severe walking difficulties [26]. By contrast, ML balance impairment can be observed in patients with atypical parkinsonisms [27] and an augmented body sway in the ML axis could help in the differential diagnosis between PD and atypical parkinsonisms [27].

Kerr et al. found that future PD fallers had not ML but AP greater postural sway when standing on a firm surface compared to nonfallers [28]. In our study, we made a comparison between PD patients without subjective BD and healthy controls, whereas in Kerr's paper the comparison is between parkinsonians fallers and nonfallers: thus, the populations taken into account are different (e.g., such patients presented freezing of gait in the cited paper). The differences found in these previous studies could indicate that there is an extensive and continuous spectrum of alterations involving both the AP and the ML balance control systems. Thus, the existence of a predominant alteration in the ML axis does not exclude a concomitant or subsequent pathological involvement of the mechanism that controls the AP body sway.

S. L. Mitchell et al. found an increase in ML sway in quite stance in Parkinsonian subjects as compared to age-matched controls. ML posturographic measures were also associated with poor performance on clinical measures of balance. For these authors, the increase in ML sway may reflect an attempt to maintain stabilizing movements during quiet stance, in order to compensate for the impaired movement in the AP direction. This notion supports the idea that ML instability could be a posturographic marker of functional balance impairment in PD [29].

The most important finding of our study is that the higher ML sway found in PD patients not complaining of BD may indicate a subclinical index of impaired balance. The maintenance of stability in ML direction requires active control, while the AP stability requires passive control [30]. In quite stance, AP balance is under ankle (plantar/dorsiflexor) control, whereas ML balance is under hip (abductor/adductor) control [31]. Both ankle and hip strategies contribute to the net balance control in different way [31]. In the ML direction, the two strategies reinforce, whereas in the AP direction the ankle mechanism must cancel most of the inappropriate contribution by the hip load/unload mechanism [31]. Basal ganglia, in particular the substantia nigra and its projections to the upper dorsal brainstem, help to optimize muscle tone for the balance control. This mechanism is disrupted in PD. As a consequence, an increased stiffness in ankle muscles in Parkinsonians has been demonstrated [32], which can explain higher sway in ML axis with a poor activation of muscles facilitating the AP sway, thus favoring nonphysiological ML oscillations.

Finally, we found that the OE condition is only slightly better than the OEC in differentiating between groups. It is known that the PD patients have more difficulties compared to healthy subjects in performing dual task. Performing a cognitive (like counting) or motor task during standing increases postural sway, particularly in PD patients. However, switching from quiet stance to concurrent task conditions in which subject's attention is diverted showed similar rates of change in both control and PD patients [33]. It can indicate that there is no significant contribution from attentional strategies in maintaining balance in PD. Further, our findings with OEC can be explained considering that postural sway increases in this condition also in healthy individuals.

### 4.2. Implications for Balance Rehabilitation in PD

The finding of a pathological total and ML body sway in PD patients not complaining of BD indicates the possibility of using posturography in order to develop a specific rehabilitation program for balance disorders.

It is possible to use different strategies to counteract the total and ML pathological sway. Clinicians could use exercises associated with cues or feedback (visual and/or auditory) in order to improve the mechanisms directed to this goal [34, 35].

PD is typically an asymmetrical disease. Previous studies using posturography have shown that balance control, which is an intuitively symmetrical task, can also be asymmetrically affected in PD [36]. This aberrant control determines that one leg produces more force than the other one in order to keep the body upright. The upright position in quiet stance, the mechanisms of balance control, and the gait initiation are correlated with each other. Indeed, gait initiation involves motor asymmetry, because the step leg must be unloaded, thereby introducing an asymmetric ML weight distribution [37]. Since we found an alteration in the ML balance control system in Parkinsonians not complaining of BD, we argue that the altered weight distribution between the two sides of the body can contribute to the increase in ML body sway in PD. The ability to transfer body weight from one leg to the other is a basic aspect of human locomotion. The transfer requires postural adjustments, necessary for both the gait and the maintenance of balance [38]. An asymmetric force between the left and the right leg can determine asymmetry in balance control in PD [39]. Thus, a balance rehabilitation program can also include a weight-shift training to improve the asymmetry in the body sway, as already demonstrated in chronic stroke patients [38].

Impaired proprioception is another contributing factor to chronic BD in PD [40]. It is known that quiet standing predominantly depends on somatosensory processing, with proprioception as the principal component [41]. Basal ganglia neurons have many proprioceptive receptive fields and this explain why proprioceptive deficits may contribute to the impaired postural and balance control in PD. Proprioceptive dysfunction in PD has been shown to be responsible for postural instability not only impairing adaption to a changing base-of-support [42], but also reducing the perception of trunk and surface orientation and postural sway in stance. These factors can partially explain why Parkinsonian patients show smaller limits of stability [43] and higher ML sway in our and in other previous studies in PD [44]. On this basis, we suggest that also a proprioceptive-motor training rehabilitation program can significantly influence balance and produce improvements in BD.

Furthermore, as previously argued, the AP balance control mechanism can be also impaired when ML abnormal sway is present. As a matter of fact, PD patients lack the modification of postural muscle synergies required to forward displace their CoP, indicating high stiffness in ankle muscles [45]. Ankle mechanisms dominate during normal stance, especially in the AP plane [46], and the increased ankle muscle stiffness contributes to balance control impairment. Kinematics showed a reduction of the range of motion in the hip, knee, and ankle joints [47]. Since the ankle plays an important role in AP balance control, a rehabilitation strategy should include stretching exercises intended to minimize the stiffness of the ankle. Moreover, narrow stance, that is typical in PD, decreased the role of the ankle and increased the role of hip mechanisms in the AP plane [46]. Therefore, in order to maintain physiological dynamic between the AP and the ML mechanisms of balance control, clinicians should immediately intervene to broaden the base of support of patients with focused exercises.

We argue that BD in Parkinsonian patients can be disclosed before the appearance of symptomatic manifestations using PP. We believe that a specific early rehabilitation treatment aimed at acting on altered ML balance control mechanism has to include (i) stretching exercises intended to minimize the stiffness of the ankle, (ii) a weight-shift training program, to improve the asymmetry in balance control, (iii) a proprioceptive-motor training, (iv) interventions to broaden the base of support, and (v) a balance training based on visual and auditory feedback.

### 4.3. Study Limitations

This study has some limitations: in order to correlate the posturographic data with the clinical outcome and to determine their prognostic value, a prospective study should be designed, performing the posturography in a cohort of early PD patients and then checking the occurrence of clinical balance troubles through the disease evolution. Furthermore, it could be useful to perform also a retrospective assessment on PD subjects divided into two groups of fallers and nonfallers. Another criticism derives from the observation that the measurements of postural stability during static and dynamic tasks have some limitations when used in a clinical setting, because patients' self-perceived risk of falling seems more reliable than objective evaluations of BD [48]. However, this may be misleading in PD patients in light of their awareness and wrong priority problems [49].

## 5. Conclusions

We have demonstrated the high sensitivity of quantifiable posturographic parameters in detecting balance disruption in PD patients not complaining of BD. These results are consistent with the literature and confirm the alteration of the complex mechanism of balance control in PD. We believe that the clinical utilization of these parameters can be useful to identify PD patients at risk of disabling BD. The obtained data can represent a possible starting point to develop specific rehabilitation treatments for balance dysfunction and BD in PD.

## Figures and Tables

**Table 1:** Demographic and clinical characteristics of patients and controls.

Variable	All patients (*N* = 29)	Controls (*N* = 12)	*p* value^∗^	Patients at low risk of fall (*N* = 23)	*p* value^†^
Sex (M/F)	12/17	3/9	0.480	10/13	0.463
Age (years)	69.2 ± 8.8	66.5 ± 6.3	0.322	67.8 ± 8.5	0.649
Weight (kilograms)	75.8 ± 10.4	73.4 ± 9.6	0.48	75.9 ± 11.3	0.52
Height (centimeters)	173.7 ± 8.2	172.6 ± 9.0	0.69	174.4 ± 7.3	0.51
Education (years)	10.06 ± 4.8	10.5 ± 5.4	0.80	10.1 ± 4.7	0.86
Disease duration (years)	10.6 ± 5.2			11.2 ± 5.0	
UPDRS III	18.6 ± 5.5			18.1 ± 5.7	
Side of disease onset (L/R)	18/11			12/11	
L-DOPA equivalent (mg/day)	742.2 ± 336.4			792.2 ± 340.8	

^∗^ *p* value for the comparison between all patients and controls.

^†^ *p* value for the comparison between patients at low risk of fall and controls.

UPDRS III: Unified Parkinson's Disease Rating Scale, part III, ON state.

**Table 2:** Values of BBS and posturographic parameters observed in patients and controls.

Variable	All patients (*N* = 29)	Controls (*N* = 12)	*p* value^∗^	Patients at low risk of fall (*N* = 23)	*p* value^†^
BBS	46.3 ± 8.4	54.3 ± 1.7	0.002	49.6 ± 4.5	0.002
AP SD OE (mm)	5.7 ± 3.0	5.9 ± 2.9	0.852	5.4 ± 2.3	0.596
ML SD OE (mm)	5.3 ± 2.6	3.4 ± 0.8	0.019	4.8 ± 2.3	0.050
Area ellipse OE (mm^2^)	601.6 ± 581.6	373.5 ± 202.2	0.195	510.0 ± 446.9	0.324
Total SD OE (mm)	15.1 ± 14.1	3.0 ± 1.6	0.005	14.1 ± 14.5	0.013
AP SD OEC (mm)	5.7 ± 3.8	5.4 ± 1.6	0.813	5.5 ± 3.7	0.957
ML SD OEC (mm)	4.8 ± 3.4	3.3 ± 1.3	0.172	4.5 ± 3.5	0.283
Area ellipse OEC (mm^2^)	477.1 ± 493.2	321.4 ± 179.7	0.297	385.7 ± 376.4	0.583
Total SD OEC (mm)	13.1 ± 13.6	3.5 ± 2.1	0.020	14.0 ± 14.3	0.017

^∗^ *p* value for the comparison between all patients and controls.

^†^ *p* value for the comparison between patients at low risk of fall and controls.

BBS: Berg Balance Scale; UPDRS III (ON state): Unified Parkinson's Disease Rating Scale III; OE: open eyes; OEC: open eyes counting; AP SD OE: anteroposterior standard deviation of trunk sway with open eyes; ML SD OE: mediolateral standard deviation of trunk sway with open eyes; Total SD OE: total standard deviation of trunk sway with open eyes; AP SD OEC: anteroposterior standard deviation of trunk sway with open eyes counting; ML SD OEC: mediolateral standard deviation of trunk sway with open eyes counting; Total SD OEC: total standard deviation of trunk sway with open eyes counting.

**(a):** 

	Age	BBS	UPDRS III	AP SD OE	ML SD OE	Area ellipse OE	Total SD OE	AP SD OEC	ML SD OEC	Area ellipse OEC	Total SD OEC
Age	1.00	−0.46 (*p* = 0.011)	0.28 (*p* = 0.145)	−0.08 (*p* = 0.679)	0.32 (*p* = 0.089)	0.05 (*p* = 0.778)	−0.35 (*p* = 0.060)	0.22 (*p* = 0.259)	0.31 (*p* = 0.107)	0.40 (*p* = 0.037)	−0.41 (*p* = 0.029)
BBS	−0.46 (*p* = 0.011)	1.00	−0.37 (*p* = 0.050)	−0.13 (*p* = 0.502)	−0.49 (*p* = 0.007)	−0.32 (*p* = 0.093)	−0.11 (*p* = 0.562)	−0.27 (*p* = 0.153)	−0.30 (*p* = 0.110)	−0.53 (*p* = 0.004)	0.07 (*p* = 0.711)
UPDRS III	0.28 (*p* = 0.145)	−0.37 (*p* = 0.050)	1.00	0.04 (*p* = 0.824)	0.20 (*p* = 0.309)	0.19 (*p* = 0.343)	0.02 (*p* = 0.906)	0.16 (*p* = 0.408)	0.30 (*p* = 0.117)	0.30 (*p* = 0.129)	−0.06 (*p* = 0.746)
AP SD OE	−0.08 (*p* = 0.679)	−0.13 (*p* = 0.502)	0.04 (*p* = 0.824)	1.00	0.44 (*p* = 0.016)	0.86 *p* < 0.001	0.14 (*p* = 0.476)	0.46 (*p* = 0.012)	0.22 (*p* = 0.256)	0.37 (*p* = 0.051)	−0.13 (*p* = 0.517)
SD ML OE	0.32 (*p* = 0.089)	−0.49 (*p* = 0.007)	0.20 (*p* = 0.309)	0.44 (*p* = 0.016)	1.00	0.79 *p* < 0.001	0.04 (*p* = 0.849)	0.67 *p* < 0.001	0.80 *p* < 0.001	0.74 *p* < 0.001	−0.02 (*p* = 0.898)
Area ellipse OE	0.05 (*p* = 0.778)	−0.32 (*p* = 0.093)	0.19 (*p* = 0.343)	0.86 *p* < 0.001	0.79 *p* < 0.001	1.00	0.13 (*p* = 0.518)	0.71 *p* < 0.001	0.58 *p* < 0.001	0.63 *p* < 0.001	−0.11 (*p* = 0.583)
Total SD OE	−0.35 (*p* = 0.060)	−0.11 (*p* = 0.562)	0.02 (*p* = 0.906)	0.14 (*p* = 0.476)	0.04 (*p* = 0.849)	0.13 (*p* = 0.518)	1.00	−0.06 (*p* = 0.745)	−0.15 (*p* = 0.423)	−0.01 (*p* = 0.974)	0.86 *p* < 0.001
AP SD OEC	0.22 (*p* = 0.259)	−0.27 (*p* = 0.153)	0.16 (*p* = 0.408)	0.46 (*p* = 0.012)	0.67 *p* < 0.001	0.71 *p* < 0.001	−0.06 (*p* = 0.745)	1.00	0.57 (*p* = 0.001)	0.89 *p* < 0.001	−0.17 (*p* = 0.392)
ML SD OEC	0.31 (*p* = 0.107)	−0.30 (*p* = 0.110)	0.30 (*p* = 0.117)	0.22 (*p* = 0.256)	0.80 *p* < 0.001	0.58 *p* < 0.001	−0.15 (*p* = 0.423)	0.57 (*p* = 0.001)	1.00	0.79 *p* < 0.001	−0.13 (*p* = 0.485)
Area ellipse OEC	0.40 (*p* = 0.037)	−0.53 (*p* = 0.004)	0.30 (*p* = 0.129)	0.37 (*p* = 0.051)	0.74 *p* < 0.001	0.63 *p* < 0.001	−0.01 (*p* = 0.974)	0.89 *p* < 0.001	0.79 *p* < 0.001	1.00	−0.12 (*p* = 0.550)
Total SD OEC	−0.41 (*p* = 0.029)	0.07 (*p* = 0.711)	−0.06 (*p* = 0.746)	−0.13 (*p* = 0.517)	−0.02 (*p* = 0.898)	−0.11 (*p* = 0.583)	0.86 *p* < 0.001	−0.17 (*p* = 0.392)	−0.13 (*p* = 0.485)	−0.12 (*p* = 0.550)	1.00

BBS: Berg Balance Scale; UPDRS III: Unified Parkinson's Disease Rating Scale, part III; OE: open eyes; OEC: open eyes counting; AP SD OE: anteroposterior standard deviation of trunk sway with open eyes; ML SD OE: mediolateral standard deviation of trunk sway with open eyes; Total SD OE: total standard deviation of trunk sway with open eyes; AP SD OEC: anteroposterior standard deviation of trunk sway with open eyes counting; ML SD OEC: mediolateral standard deviation of trunk sway with open eyes counting; Total SD OEC: total standard deviation of trunk sway with open eyes counting.

**(b):** 

	Age	BBS	AP SD OE	ML SD OE	Area ellipse OE	Total SD OE	AP SD OEC	ML SD OEC	Area ellipse OEC	Total SD OEC
Age	1.00	−0.68 (*p* = 0.016)	0.05 (*p* = 0.874)	0.21 (*p* = 0.515)	0.06 (*p* = 0.841)	−0.01 (*p* = 0.986)	−0.31 (*p* = 0.330)	0.08 (*p* = 0.811)	−0.24 (*p* = 0.462)	−0.04 (*p* = 0.891)
BBS	−0.68 (*p* = 0.016)	1.00	−0.10 (*p* = 0.753)	−0.38 (*p* = 0.227)	−0.18 (*p* = 0.574)	0.19 (*p* = 0.546)	−0.16 (*p* = 0.625)	−0.22 (*p* = 0.500)	−0.07 (*p* = 0.834)	0.07 (*p* = 0.827)
AP SD OE	0.05 (*p* = 0.874)	−0.10 (*p* = 0.753)	1.00	−0.06 (*p* = 0.849)	0.91 *p* < 0.001	0.28 (*p* = 0.382)	0.38 (*p* = 0.218)	−0.11 (*p* = 0.732)	0.06 (*p* = 0.841)	0.32 (*p* = 0.314)
SD ML OE	0.21 (*p* = 0.515)	−0.38 (*p* = 0.227)	−0.06 (*p* = 0.849)	1.00	0.31 (*p* = 0.321)	−0.04 (*p* = 0.903)	0.36 (*p* = 0.250)	0.56 (*p* = 0.060)	0.42 (*p* = 0.176)	−0.25 (*p* = 0.442)
Area ellipse OE	0.06 (*p* = 0.841)	−0.18 (*p* = 0.574)	0.91 *p* < 0.001	0.31 (*p* = 0.321)	1.00	0.30 (*p* = 0.340)	0.58 (*p* = 0.047)	0.20 (*p* = 0.528)	0.30 (*p* = 0.336)	0.31 (*p* = 0.328)
Total SD OE	−0.01 (*p* = 0.986)	0.19 (*p* = 0.546)	0.28 (*p* = 0.382)	−0.04 (*p* = 0.903)	0.30 (*p* = 0.340)	1.00	0.45 (*p* = 0.144)	0.63 (*p* = 0.030)	0.65 (*p* = 0.022)	0.70 (*p* = 0.011)
AP SD OEC	−0.31 (*p* = 0.330)	−0.16 (*p* = 0.625)	0.38 (*p* = 0.218)	0.36 (*p* = 0.250)	0.58 (*p* = 0.047)	0.45 (*p* = 0.144)	1.00	0.68 (*p* = 0.014)	0.88 *p* < 0.001	0.43 (*p* = 0.165)
ML SD OEC	0.08 (*p* = 0.811)	−0.22 (*p* = 0.500)	−0.11 (*p* = 0.732)	0.56 (*p* = 0.060)	0.20 (*p* = 0.528)	0.63 (*p* = 0.030)	0.68 (*p* = 0.014)	1.00	0.90 *p* < 0.001	0.41 (*p* = 0.184)
Area ellipse OEC	−0.24 (*p* = 0.462)	−0.07 (*p* = 0.834)	0.06 (*p* = 0.841)	0.42 (*p* = 0.176)	0.30 (*p* = 0.336)	0.65 (*p* = 0.022)	0.88 *p* < 0.001	0.90 *p* < 0.001	1.00	0.45 (*p* = 0.142)
Total SD OEC	−0.04 (*p* = 0.891)	0.07 (*p* = 0.827)	0.32 (*p* = 0.314)	−0.25 (*p* = 0.442)	0.31 (*p* = 0.328)	0.70 (*p* = 0.011)	0.43 (*p* = 0.165)	0.41 (*p* = 0.184)	0.45 (*p* = 0.142)	1.00

BBS: Berg Balance Scale; OE: open eyes; OEC: open eyes counting; AP SD OE: anteroposterior standard deviation of trunk sway with open eyes; ML SD OE: mediolateral standard deviation of trunk sway with open eyes; Total SD OE: total standard deviation of trunk sway with open eyes; AP SD OEC: anteroposterior standard deviation of trunk sway with open eyes counting; ML SD OEC: mediolateral standard deviation of trunk sway with open eyes counting; Total SD OEC: total standard deviation of trunk sway with open eyes counting.
